# Three Patients Needing High Doses of Valproic Acid to Get Therapeutic Concentrations

**DOI:** 10.1155/2015/542862

**Published:** 2015-04-27

**Authors:** James Jackson, Betsy McCollum, Judy Ognibene, Francisco J. Diaz, Jose de Leon

**Affiliations:** ^1^Department of Psychiatry, College of Medicine, University of Kentucky, Lexington, KY 40509, USA; ^2^Pharmacy, Eastern State Hospital, Lexington, KY 40511, USA; ^3^Apalachee, Inc., Eastside Psychiatric Hospital, Tallahassee, FL 32308, USA; ^4^Department of Biostatistics, The University of Kansas Medical Center, Kansas City, KS 66160, USA; ^5^University of Kentucky Mental Health Research Center, Eastern State Hospital, Lexington, KY 40511, USA

## Abstract

Valproic acid (VPA) can autoinduce its own metabolism. Cases requiring VPA doses >4000 mg/day to obtain therapeutic plasma concentrations, such as these 3 cases, have never been published. Case 1 received VPA for seizures and schizophrenia and had >50 VPA concentrations in 4 years. A high dose of 5,250 mg/day of VPA concentrate was prescribed for years but this dose led to an intoxication when switched to the enterocoated divalproex sodium formulation, requiring a normal dose of 2000 mg/day. VPA metabolic capacity was significantly higher (*t* = −9.6; df = 6.3, *p* < 0.001) during the VPA concentrate therapy, possibly due to autoinduction in that formulation. Case 2 had VPA for schizoaffective psychosis with 10 VPA concentrations during an 8-week admission. To maintain a VPA level ≥50 *μ*g/mL, VPA doses increased from 1500 to 4000 mg/day. Case 3 had tuberous sclerosis and epilepsy and was followed up for >4 years with 137 VPA concentrations. To maintain VPA concentrations ≥50 *μ*g/mL, VPA doses increased from 3,375 to 10,500 mg/day. In Cases 2 and 3, the duration of admission and the VPA dose were strongly correlated (*r* around 0.90; *p* < 0.001) with almost no change after controlling for VPA concentrations, indicating progressive autoinduction that increased with time.

## 1. Introduction

Valproic acid (VPA) is a classic antiepileptic drug (AED) and has been a major pharmaceutical tool in the management of a range of psychiatric and neurological diseases since the 1960s [1]. VPA is currently approved in the US for the treatment of several types of epilepsy, bipolar disorder, and migraine prophylaxis. The maximum recommended doses for epilepsy or bipolar disorder are 60 mg/kg/day [2]. It is rare to see patients taking >4000 mg/day. Moreover, therapeutic drug monitoring (TDM) is frequently used to establish and track VPA doses. Neurologists frequently use a therapeutic range of 50–100 *μ*g/mL for epilepsy [3]. The therapeutic range in bipolar disorder is not very well established but some reviews recommend up to 125 *μ*g/mL for mania [2].

In spite of decades of use, VPA metabolism is not completely understood [3–8]. VPA primarily undergoes hepatic metabolism, while <5% is eliminated and unchanged in the urine. The major mechanisms of hepatic metabolism are the uridine diphosphate glucuronosyltransferases (UGTs; 40%) and *β*-oxidation as a fatty acid (30%), with the cytochrome P450 (CYP; including CYP2C9, CYP2C19, and CYP2A6) as a minor component in the metabolic process. At low doses, *β*-oxidation may be the most important pathway, while at therapeutic doses glucuronidation may be more important [3–8]. Many UGTs appear to be involved in valproate glucuronidation including UGT1A3, UGT1A4, UGT1A6, UGT1A9, and UGT2B7, along with the primarily intestinal UGT1A8 and UGT1A10 [9].

Regarding the potential of causing drug-drug interactions (DDIs), VPA was traditionally considered to be a moderate inhibitor of several enzymes including CYP2C9, epoxide hydroxylase, several UGTs, and the N-glucosidation pathway of phenobarbital [4]. First, rat studies suggested that VPA may autoinduce its own glucuronidation [10]. The first clinical suggestions that VPA may autoinduce its own metabolism were from studies focused on felbamate [11] and lamotrigine [12]. More recent studies have been able to demonstrate that VPA induces (1) its own metabolism by inducing *β*-oxidation (prospective study) [13]; (2) CYP3A4 and P-gp gene expression (in vitro study) [14]; (3) possible UGT1A1 in a patient taking irinotecan (which has an active metabolite, SN-38, metabolized by UGT1A1) [15]; (4) aripiprazole metabolism to a mild degree (prospective study) [16]; (5) olanzapine metabolism (case series [17], TDM [18], a statistical model of TDM-DDI studies [19], and a prospective DDI study) [20]; (6) clozapine metabolism (case series [21, 22], a prospective case report [23], and statistical models of TDM-DDI studies [24, 25]); and (7) vitamin D metabolism in an in vitro study [26]. In summary, VPA may be like other AEDs, such as oxcarbazepine or topiramate, and act as a mild inducer for some metabolic enzymes and behave as a clinically relevant inhibitor of other metabolic enzymes.

The following 3 cases, accumulated by the senior author in the last 20 years of his clinical practice, describe 3 patients needing high doses of VPA to reach and maintain therapeutic concentrations ≥50 *μ*g/mL. Recent advances in pharmacokinetic knowledge have allowed us to offer a hypothesis about these patients' needs for such high VPA doses to reach therapeutic concentrations. On February 19, 2015, we conducted a PubMed search using the words “valproic acid and (high dose or high dosage)” limiting them to title or abstract, human participants, and case reports, which provided 33 articles. None of these 33 articles described the therapeutic use of VPA doses >4000 mg/day. We decided to publish these cases because it is very likely that other clinicians have seen similar patients but did not write about them.

## 2. Methods

### 2.1. Clinical Setting

The senior author collected these 3 cases during the last 20 years while working as a clinician and/or consultant in the public mental health system in Kentucky, USA. During 8 of those years, he managed a 30-bed treatment-refractory unit for psychotic patients in a state hospital with approximately 1600 admissions/year. He acquired Cases 1 and 3 in that setting. For 14 years he has also acted as a consultant for difficult cases, including those needing high doses of psychiatric medications in 4 state hospitals for severe mentally ill patients, 4 hospitals for adults with intellectual disabilities, and 2 nursing homes. Case 2 was collected as a consultant for another psychiatrist (third author). In the senior author's experience, VPA is probably the most frequently prescribed psychotropic drug in the public mental health system facilities of Kentucky, with more than 1000 patients every year treated with VPA at state facilities. The 3 patients in this paper participated in pharmacogenetic studies after written informed consent forms were signed by them and/or their guardians.

### 2.2. TDM

These 3 cases contain TDM information that was collected for clinical purposes many years ago. The VPA TDM was done by immunoassay in the same psychiatric hospital using the same clinical laboratory at the hospital; levels were taken as trough concentrations (early AM before taking medications) and after reaching steady state. The VPA concentrations were measured in *μ*g/mL, which is the same as mg/L.

In the last 10 years, the senior author has increasingly used the pharmacological concept of concentration-to-dose ratio (C/D ratio) to study TDM in order to personalize the prescription of psychiatric medications in psychopharmacology. The C/D ratio is a measure of the ability to eliminate the drug and is influenced by genetic, personal, and environmental factors. Inducers decrease the C/D ratio and inhibitors increase the C/D ratio. In comparing individuals taking the same drug, a very low C/D ratio indicates an individual with very fast metabolism, while a very high C/D ratio indicates one with very slow metabolism. Each drug is different and unique and has its own normal ranges for C/D ratios determined by its own bioavailability and elimination from the body.

The senior author has used the C/D ratio to interpret TDM of clobazam [27], clozapine, and risperidone [28]. These 3 drugs, like the majority of drugs used in neuropsychopharmacology, follow linear kinetics. A linear relationship exists between typical doses and plasma concentrations. This means that the relationship between concentration and dose is stable; it does not change with different doses and concentrations, and the drug C/D ratio is constant in the same patient as long as there are no changes in environmental or personal variables.

VPA TDM, unfortunately, is a little more complicated since it does not follow linear kinetics. The relationship between VPA dose and total concentration is nonlinear; the concentration does not increase proportionally with the dose but increases to a lesser extent due to saturable plasma-protein binding [29]. As the VPA C/D ratio is not constant and changes with different doses and concentrations, one needs to further interpret VPA C/D ratios in the context of a set of concentrations or set of doses.

The senior author has started using VPA C/D ratios in his teaching and clinical practice in the last 3 years and is not aware of any published article using them. Therefore, as the reader may not be familiar with the use of C/D ratios for valproate VPA, this section provides a short introduction. Based on experience as a lecturer, the senior author has figured out that VPA C/D ratios have values too low to be easily understood by physicians, who do not tend to be particularly strong in mathematics. To more easily understand the VPA C/D values, they are also presented with a value obtained by multiplying by 1000. To understand this, let us assume that a VPA dose of 2000 mg/day provides a total VPA concentration of 100 *μ*g/mL. Therefore the C/D ratio in this patient is 100/2000 or 0.05. The C/D ratio multiplied by 1000 would be 50, an easier number to grasp.

Next, we can use a published case of VPA toxicity [29] to explain the interpretation of VPA C/D ratio for clinical use. To simplify, Table 1 presents only the VPA data including C/D ratios using total VPA concentrations. This patient has relatively narrow variations, with a C/D ratio multiplied by 1000 ranging between 112 and 132 (Table 1). These values are normal in the experience of the senior author. Moreover, these values follow the usual pattern of VPA C/D ratios. In low doses, in this case 500 mg/day, the C/D ratio multiplied by 1000 ranged from 128 to 132. In high doses, in this case 1000 mg/day, the C/D ratio multiplied by 1000 was 112, indicating a faster metabolism at 1000 mg/day than at the 500 mg/day dose. At higher VPA doses, plasma proteins such as albumin are saturated by VPA and the percentage of free (unbound) VPA concentration increases. Since free VPA is the entity that is metabolized, the higher the percentage of free concentration is, the faster the VPA is metabolized. In the experience of the senior author, approximately 90% of VPA on average is carried by plasmatic proteins and 10% is free at standard therapeutic concentrations. With higher concentrations, the relative concentrations of free VPA increase (e.g., 85% bound and 15% free), and with low concentrations it decreases (e.g., 95% bound and 5% free). These percentages of free VPA concentration are also influenced by (1) the plasma concentration of albumin and other plasmatic proteins, (2) plasma concentrations of some endogenous compounds that may bind to the proteins (e.g., bilirubin), and (3) the presence of other drugs (e.g., aspirin), which may also compete for plasmatic protein binding [29].

Other examples of VPA C/D ratio are calculated using published data [30, 31]. A case [30] of a possible VPA adverse drug reaction (ADR) occurred in a patient who had a VPA concentration 78 *μ*g/mL at a dose of 1500 mg/day (C/D ratio = 0.520 calculated by 78/1500). The VPA C/D ratio multiplied by 1000 was 520. In a study of VPA TDM [31], 14 females had an average VPA concentration of 76 *μ*g/mL with an average maintenance dose of 629 mg/day (C/D ratio = 0.121 calculated by 76/629) and a VPA C/D ratio multiplied by 1000 of 121. In the same study, 23 males had an average VPA concentration of 70 *μ*g/mL with an average maintenance dose of 617 mg/day (C/D ratio = 0.113 calculated by 70/617) with a VPA C/D ratio multiplied by 1000 of 113.

These 3 cases needing VPA doses >4000 mg/day to obtain VPA therapeutic concentrations had very low mean C/D ratios multiplied by 1000: in the 20s or lower, possibly due to VPA autoinduction.

### 2.3. Scale

For this paper, we have completed the Drug Interaction Probability Scale (DIPS) [32] for each of the 3 cases.

### 2.4. Statistics

Available data for each of the 3 cases was dictated by clinical care; many years later we tried to use statistical techniques to accommodate the available data and the questions asked in each case. The Statistical Package for the Social Sciences (SPSS 22) was used to analyze the data. In Case 1 an independent sample *t*-test compared mean VPA C/D ratios from the two VPA formulations, one probably accompanied with autoinduction and the other not. Pearson correlations between VPA dose and day of admission were used in Cases 2 and 3 to demonstrate that higher doses were needed to keep the VPA concentrations therapeutic over time, which is consistent with a pattern of autoinduction. Partial correlations were used to demonstrate that VPA concentrations did not explain the correlation between VPA dose and day of admission.

## 3. Case Presentations

### 3.1. Case 1

A Caucasian male was followed up for more than 4 years between the ages of 30 and 34 years. His initial weight was 85 Kg. He smoked 10 cigarettes per day. His psychosis started when he was 12 years old and had been refractory to treatment for many years at the time of admission. The patient had three seizures of unknown origin in the 3 months before coming under the care of the senior author. Academic neurologists had examined him at least twice. All tests, including a CT scan of the head, were negative. After a careful review of all records, it was the impression of the senior author that at least 1 or 2 of the 3 seizures might have been associated with rapid withdrawal of high doses of benzodiazepines, particularly temazepam, used as PRNs. He arrived at the unit with four antiepileptic medications in his regimen: carbamazepine, phenytoin, diazepam, and VPA. These medications had been progressively added by the neurology consultants. The blood levels of all four of these medications were subtherapeutic. After several months the senior author was able to change the regimen to VPA only, without the patient having any seizures during his long admission. The VPA treatment is described in detail in Table 2. To avoid too much irrelevant information, day 164 of admission was selected as day 1 to describe the time-course of VPA treatment. The senior author was surprised that this patient needed 5250 mg/day of VPA concentrate to get therapeutic VPA concentrations. After more than 3.5 years of admission, the patient was finally stabilized on clozapine at 700 mg/day, his psychotic symptoms had greatly improved, and he was getting ready to be discharged. However, he then began to complain about the taste of the VPA concentrate in his mouth. With the assumption of bioequivalent formulations and dosing, the patient was switched to divalproex sodium at a total daily dose of 5250 mg/day. This led to an unexpected VPA intoxication despite the absence of any other medication changes. This unexpected outcome left the senior author perplexed and led him to a brief preliminary publication on VPA concentrations during the last 3 months of admission [33], despite having no pharmacological explanation at that time. Table 2 presents a comprehensive list of the VPA concentrations during most of 4 years, as well as the corresponding VPA C/D ratios. While the patient was being maintained on 5250 mg/day of VPA concentrate for many years, the VPA C/D ratio multiplied by 1000 yielded values ranging from 10 to 21 with a mean of 15. Divalproex sodium treatment yielded VPA C/D values which, multiplied by 1000, ranged from 28 to 48, with a mean of 39. The means of 19 and 39 were found to be significantly different (Table 2, footnote 1), indicating that the patient's ability to metabolize VPA was significantly higher on VPA concentrate than on divalproex sodium.

It is likely that this case presentation reflects the result of VPA autoinduction only present during VPA concentrate versus no autoinduction during divalproex sodium use. The DIPS score was 6, which corresponds to a probable drug interaction in this case (scored as 1 point each for questions 2, 3, 4, 7, 8, and 9).

### 3.2. Case 2

Patient 2 was a 66-year-old Caucasian male nonsmoker with a 26-year history of bipolar disorder and a history of polysubstance abuse (alcohol and cocaine). His initial weight was 90 Kg. He had 7 prior psychiatric admissions. On presentation the patient met the criteria for hypomanic episode with psychotic features. He had a positive viral panel for hepatitis C and his Hep C RNA PCR was positive, but his liver function profile and metabolic panel were unremarkable. Sublingual asenapine 20 mg/day, one of his outpatient medications, was restarted by the third author on hospital day 2 in addition to VPA 1000 mg/day, which the patient had not received as an outpatient. Other medications are described in Table 3. The patient did not experience any signs of VPA toxicity as the dose was increased during his admission. However, he remained hypomanic and continued to display rapid, pressured speech and flight of ideas in addition to increased levels of energy. His VPA concentrations were monitored closely with gradual increases in his enterocoated divalproex sodium. Despite increasing his daily dose and switching to concentrate and then back to enterocoated divalproex sodium, the dose had to be progressively increased to a total of 4000 mg/day to continue to have a therapeutic response and therapeutic levels. By the time of discharge, the patient's mood had become more euthymic. He was discharged after 10 weeks of inpatient management in stable condition and with a medication regimen including VPA at 4000 mg/day. This patient was remarkable because, in order to maintain the therapeutic response and a VPA level around 70 *μ*g/mL, progressively higher doses from 1500 to 4000 mg/day were needed to the point that the last VPA C/D ratio multiplied by 1000 had a value remarkably low (at 17) while being on a high dose of 4000 mg/day and with a VPA concentration of only 67 *μ*g/mL. The mean C/D ratio multiplied by 1000 was 25 with a range from 17 to 33 (Table 3). As a matter of fact, in spite of the limited number of VPA concentrations, there was a very strong correlation (*r* = 0.97; *p* < 0.001) between the number of days of admission and the dose, indicating that, with an increasing length of admission, the dose increased, and this high value remained after correcting for VPA concentrations using a partial correlation (*r* = 0.96; *p* < 0.001) (Table 3, footnote 1). This is compatible with a progressive autoinduction; as the time after admission increased, it was necessary to increase the VPA dose. The patient was discharged on 4000 mg/day; we do not know what happened after discharge, except that the patient had no relapses for 2 years after this admission.

Considering the need for higher doses to maintain the same VPA concentration, the DIPS score was 5 (scoring 1 point each for questions 2, 3, 4, 7, and 8), indicating a “probable” relationship between VPA and autoinduction activity.

### 3.3. Case 3

Patient 3 was a Caucasian male nonsmoker with a history of tuberous sclerosis, which manifested as seizure disorder and mental retardation. His initial weight was 71 Kg. He had been an inpatient at a state psychiatric hospital for 22 years before the senior author started to manage his treatment (day 1) when the patient was 48 years old. Two years later he developed a right renal mass, which was highly suspicious for renal cell carcinoma on biopsy. It was followed by a right-sided nephrectomy and cholecystectomy (day 860). However, the final pathology suggested that the mass was actually angiomyolipoma, which is common if there is renal involvement in patients with tuberous sclerosis. Thorough investigation for left-sided pathology indicated that his left kidney was functioning well with stable masses present on CT. From day 1322, the patient progressively worsened with new-onset episodes of ataxia and confusion. High clinical suspicion for a brain mass led the senior author to investigate with MRI. The results indicated that the patient had a mass in the right lateral ventricle “suggestive of giant cell astrocytoma” and that “foci of enhancement at the gray-white junction and numerous signal abnormalities in the skull suggest the possibility of metastatic disease.” Giant cell astrocytomas occur in 6–14% of patients with tuberous sclerosis and should be suspected when there is a new focal deficit, signs of increased intracranial pressure, or unexplained behavioral changes. After day 1420, when the brain tumor was diagnosed, we started trying to reduce the VPA dose and manage it on the basis of the patient's comfort rather than the VPA concentration level.

During the long follow-up of more than 4 years (almost 1500 days) described in Table 4, the patient had therapeutic levels of phenytoin initially with 500 mg/day and at the end with 400 mg/day. VPA showed a completely different pattern, requiring a progressive increase in the dose to keep VPA therapeutic concentrations >50 *μ*g/mL. On day 1 the VPA dose was 3375 mg/day, on day 400 it was 5000 mg/day, on day 943 it was 9000 mg/day, and on day 1029 it was 10500 mg/day, which was maintained until it was clear that his physical deterioration was due to a brain tumor. At that time other medications were discontinued, only leaving phenytoin and a lower VPA dose (Table 4).

Despite the necessity of increasing the VPA dose to this very high dosage, the phenytoin dose remained stable with normal blood levels in the therapeutic range. This suggests that the progressive increase in VPA metabolism was not associated with an increase in phenytoin metabolism. The VPA C/D ratios in this patient were extremely low, frequently lower than 10. The mean C/D ratio multiplied by 1000 for 70 therapeutic concentrations was 8 with a range from 5 to 18 (Table 3). As a matter of fact, there was a very strong correlation (*r* = 0.97; *p* < 0.001) between the number of days past admission and the dose, indicating that with an increasing length of admission, the dose increased, and this significance remained after correcting for VPA concentrations using a partial correlation (*r* = 0.89; *p* < 0.001) (Table 4, footnote 1). This is compatible with a progressive autoinduction.

Using the DIPS as in the previous cases, the total score was 5 (scoring 1 point each for questions 2, 3, 4, 7, and 8), indicating a “probable” relationship between VPA and the autoinduction of its metabolism.

## 4. Discussion

### 4.1. Limitations

These 3 cases reflect 3 extremely challenging patients who required very high doses of VPA >4000 mg/day to maintain VPA therapeutic levels. As usual in these challenging cases, the pharmacological data is somewhat limited since patients were treated with polypharmacy and went through complex changes in medications. In spite of the data limitations, we think that these cases need to be published to acknowledge that while these patients are very rare, they exist. In support of the above findings, however, the senior author was closely involved in the treatment of the 3 patients and all VPA concentrations were measured in the same lab. It is rare to see publications describe so many large measures of VPA concentrations.

The data is limited but undeniable and should indicate to any clinician that these 3 patients needed daily doses of VPA to get therapeutic VPA concentrations. The second and greater limitation in analyzing these cases is our interpretation of the data many years after they were collected—in the third case, almost 20 years. It has required that much time to accumulate evidence in the literature to understand what happened and to realize that VPA may be an inducer; in fact, it can induce its own metabolism. Therefore, although these VPA concentrations were collected prospectively and used to modify VPA doses, they were reviewed retrospectively to provide the unified hypothesis that it is possible that these high VPA doses were required because these 3 patients were unusually sensitive to VPA autoinduction. In retrospect, after having a possible pharmacological explanation now after many years, it would have been better to collect more frequent TDM data, but what is described is what is available. Although we acknowledge that the data is limited and our interpretation is even more limited, we have tried to support our interpretation through the use of statistical tests and a validated scale.

### 4.2. High Doses of VPA May Be Explained by VPA Autoinduction

We think these cases contribute to the scarce literature by indicating that clinicians must be aware that VPA may behave as an inducer. In Cases 1 and 3, patients with likely VPA autoinduction presented significant clinical challenges to the senior author, who had no idea why the patients required much higher VPA dosages to obtain therapeutic VPA concentrations. Case 2 was identified only 4 years ago when the senior author realized that VPA autoinduction was clinically relevant for some peculiar patients. As a matter of fact, Case 2 was identified by the third author, who had rarely seen similar cases. The senior author suggested to her that these cases were probably explained by autoinduction and she was able to identify this patient as a candidate for VPA autoinduction shortly after admission.

The first patient was very challenging, probably the most challenging patient in the senior author's career, even with his more than 25-year history of treating treatment-refractory patients. The patient metabolized the CYP1A2 drugs clozapine and olanzapine normally but got intoxicated on clozapine during an infection [34]. He appeared unusual because he needed 5250 mg/day of VPA concentrate for several years to get therapeutic VPA concentrations. The senior author had experience in switching many patients back and forth from VPA concentrate to divalproex sodium using the same doses and getting similar VPA concentrations. In fact, the US prescribing information from the drug manufacturer recommended and continues to recommend initiating the same total daily doses when converting a patient from VPA to divalproex sodium. A published study reported a statistically significant decrease of 14.4% (*p* = 0.001) in VPA plasma concentrations upon switching dosage forms from divalproex sodium to VPA [35]. Therefore, the senior author expected a minor increase of around 14.4% in switching from 5250 mg/day of VPA concentrate but, instead, he intoxicated the patient. The VPA concentration went from the 80s *μ*g/mL to 145 *μ*g/mL and required halving the dose to 2000 mg/day of divalproex sodium to get VPA concentrations in the 80s *μ*g/mL. In retrospect, during treatment with VPA concentrate, the VPA C/D ratio multiplied by 1000 was extremely low, at a mean of 15 and a range of 10–21, indicating an extraordinarily high capacity to metabolize VPA. This is compatible with VPA concentrate inducing its own metabolism. We propose that after 4 weeks on divalproex sodium the autoinduction was lost and the patient's metabolism normalized, with a mean VPA C/D ratio multiplied by 1000 of 39 and a range of 28 to 48. We cannot explain why this patient's VPA concentrate appeared to induce its own metabolism while divalproex sodium did not; we assume this patient had some rare genetic variation that explains his peculiar response.

The second patient had a relatively short admission (<60 days) but demonstrated a very high and significant correlation between length of admission and VPA dose. The final C/D ratio was 17, extremely low. This type of patient is probably more common than Case 1; his treating psychiatrist, the third author, has seen several similar cases in her 25-year practice in patients with severe mental illness including 20 years at this psychiatric hospital.

The third patient probably deserved a Guinness record since he was treated with 10500 mg/day for 400 days and had a VPA C/D ratio multiplied by 1000 which yielded a value less than 10 most of the time. We have the impression that progressive VPA autoinduction explained the need to go from 3450 to 10500 mg/day to achieve and maintain VPA concentrations >50 *μ*g/mL. It is possible that phenytoin treatment contributed in some way to the facilitation of VPA autoinduction, since phenytoin is a major inducer and can displace VPA from plasmatic proteins. It is remarkable that although increases in VPA dosage were needed to get therapeutic VPA concentrations, the phenytoin dosage needed to obtain therapeutic phenytoin concentrations was relatively stable throughout this time period. Phenytoin doses ranged from 500 mg/day at the beginning to 400 mg/day at the end. This minor reduction in dosage may be explained by the greater VPA concentrations at the end. VPA is an inhibitor of phenytoin metabolism. Unfortunately, 20 years ago, free VPA and phenytoin concentrations were not available at this psychiatric hospital. It would have been important to measure them [29].

### 4.3. The Pharmacological Mechanism behind VPA Induction

The induction of metabolic enzymes such as UGTs implies that the amount of these proteins increases when they are induced; this is almost always explained by increasing protein synthesis mediated by the so-called nuclear receptors (constitutive androstane, estrogen, glucocorticoid receptors, and pregnane X receptors) or other transcription factors such as aryl hydrocarbon receptors. The potent AED inducers (carbamazepine, phenytoin, and phenobarbital) bind to the pregnane X receptors. Phenytoin and phenobarbital appear to also bind to the constitutive androstane receptors. VPA is a less potent inducer but probably works in a similar way. An in vitro study suggested that VPA might also activate constitutive androstane receptor and pregnane X receptor pathways [14].

We think it is possible that these unusual patients needing very high VPA dosages have unusual genetic variants at the nuclear receptors, which made them very sensitive to VPA autoinduction.

## Figures and Tables

**Table 1:** Explaining VPA C/D ratio use with VPA concentrations from a published case [29].

Formulation	VPA dose (mg/day)	Concentration (*μ*g/mL)	C/D ratio	C/D ratio × 1000
ECDVNa	1000	112	0.112	112
ECDVNa	750	87	0.116	116
ECDVNa	500	66	0.132	132
ECDVNa	500	64	0.128	128

C/D: concentration-to-dose; ECDVNa: divalproex sodium enterocoated; VPA: valproic acid.

**Table 2:** VPA C/D ratio in Case 1.

Day	VPA dose (mg/day)	Concentration (*μ*g/mL)	C/D ratio	C/D ratio × 1000
Valproic acid concentrate (C/D ratio × 1000: mean ± SD = 15^1^ ± 2.6, range = 10–21)				
7^3^	5250	54	0.010	10
14^4^	5250	68	0.013	13
21^5^	5250	68	0.013	13
29^5^	5250	76	0.014	14
70^5^	5250	59	0.011	11
98^6^	5250	66	0.013	13
119^6^	5250	85	0.016	16
126^6^	5250	61	0.012	12
140^6^	5250	75	0.014	14
175^6^	5250	79	0.015	15
188^6^	5250	64	0.012	12
202^7^	5250	64	0.012	12
238^8^	5250	88	0.017	17
252^8^	5250	110	0.021	21
260^9^	5250	86	0.016	16
280^9^	5250	92	0.018	18
287^9^	5250	87	0.017	17
301^10^	5250	87	0.017	17
319^10^	5250	92	0.018	18
350^10^	5250	99	0.019	19
378^11^	5250	85	0.016	16
420^12^	5250	91	0.017	17
427^12^	5250	97	0.018	18
434^12^	5250	87	0.017	17
470^12^	5250	87	0.017	17
498^13^	5250	90	0.017	17
552^13^	5250	95	0.018	18
582^13^	5250	81	0.015	15
609^13^	5250	88	0.017	17
637^7^	5250	80	0.015	15
928^14^	5250	111	0.021	21
945^15^	5250	93	0.018	18
978^16^	5250	78	0.015	15
998^17^	5250	67	0.013	13
1026^18^	5250	67	0.013	13
1054^19^	5250	66	0.013	13
1082^19^	5250	71	0.014	14
1110^19^	5250	63	0.012	12
1145^20^	5250	69	0.013	13
1166^21^	5250	93	0.018	18
1202^22^	5250	89	0.017	17
1208^23^	5250	75	0.014	14
1236^24^	5250	88	0.017	17
1251^25^	5250	87	0.017	17

EC divalproex sodium (C/D ratio × 1000: mean ± SD = 39^1^ ± 6.3, range = 28–48)				
1306^25^	5250	145^2^	0.028	28
1320^25^	3750	135	0.036	36
1334^25^	3000	127	0.042	42
1373^25^	2500	120	0.048	48
1348^25^	2000	73	0.037	37
1362^25^	2000	82	0.041	41
1376^25^	2000	78	0.039	39

C/D: concentration-to-dose; EC: enterocoated; VPA: valproic acid.

^1^According to an independent *t*-test calculated with equal variance not assumed, there was significant difference (*t* = −9.6; df = 6.3, *p* < 0.001) between these two means, 15 in VPA concentrate and 39 in divalproex sodium.

^2^This VPA concentration was measured 4 weeks after switching from VPA concentrate to divalproex sodium. With a high VPA concentration at that time, the patient showed increased drowsiness.

^3^Other scheduled oral medications included benztropine 3 mg/day, gemfibrozil 1200 mg/day, propranolol 80 mg/day, and quetiapine 700 mg/day.

^4^Other scheduled oral medications included benztropine 3 mg/day, gemfibrozil 1200 mg/day, olanzapine 5 mg/day, propranolol 80 mg/day, and quetiapine 500 mg/day.

^5^Other scheduled oral medications included benztropine 3 mg/day, gemfibrozil 1200 mg/day, olanzapine 5 mg/day, and propranolol 80 mg/day.

^6^Other scheduled oral medications included benztropine 4 mg/day, gemfibrozil 1200 mg/day, olanzapine 5 mg/day, and propranolol 80 mg/day.

^7^Other scheduled oral medications included benztropine 4 mg/day, gemfibrozil 1200 mg/day, olanzapine 5 mg/day, and propranolol 60 mg/day.

^8^Other scheduled oral medications included benztropine 4 mg/day, gemfibrozil 1200 mg/day, olanzapine 2.5 mg/day, and propranolol 30 mg/day.

^9^Other scheduled oral medications included benztropine 4 mg/day, gemfibrozil 1200 mg/day, olanzapine 2.5 mg/day, and propranolol 40 mg/day.

^10^Other scheduled oral medications included benztropine 4 mg/day, gemfibrozil 1200 mg/day, olanzapine 2.5 mg/day, and propranolol 60 mg/day.

^11^Other scheduled oral medications included benztropine 4 mg/day, gemfibrozil 1200 mg/day, olanzapine 2.5 mg/day, and propranolol 120 mg/day.

^12^Other scheduled oral medications included benztropine 4 mg/day, clonidine 0.1 mg/day, gemfibrozil 1200 mg/day, olanzapine 2.5 mg/day, and propranolol 60 mg/day.

^13^Other scheduled oral medications included benztropine 4 mg/day, gemfibrozil 1200 mg/day, olanzapine 2.5 mg/day, and propranolol 60 mg/day.

^14^Other scheduled oral medications included benztropine 4 mg/day, clozapine 25 mg/day, gemfibrozil 1200 mg/day, olanzapine 10 mg/day, and propranolol 80 mg/day.

^15^Other scheduled oral medications included benztropine 4 mg/day, clozapine 300 mg/day, gemfibrozil 1200 mg/day, olanzapine 10 mg/day, and propranolol 80 mg/day.

^16^Other scheduled oral medications included benztropine 4 mg/day, clozapine 600 mg/day, gemfibrozil 1200 mg/day, olanzapine 10 mg/day, and propranolol 80 mg/day.

^17^Other scheduled oral medications included benztropine 4 mg/day, clozapine 400 mg/day, gemfibrozil 1200 mg/day, olanzapine 10 mg/day, and propranolol 80 mg/day.

^18^Other scheduled oral medications included benztropine 4 mg/day, clozapine 700 mg/day, gemfibrozil 1200 mg/day, olanzapine 10 mg/day, and propranolol 80 mg/day.

^19^Other scheduled oral medications included benztropine 4 mg/day, clozapine 800 mg/day, gemfibrozil 1200 mg/day, and propranolol 80 mg/day.

^20^Other scheduled oral medications included atorvastatin 10 mg/day, benztropine 4 mg/day, clozapine 800 mg/day, and propranolol 80 mg/day.

^21^Other scheduled oral medications included atorvastatin 20 mg/day, benztropine 4 mg/day, clozapine 800 mg/day, and propranolol 80 mg/day.

^22^Other scheduled oral medications included atorvastatin 20 mg/day, clozapine 700 mg/day, docusate 250 mg/day, and propranolol 80 mg/day.

^23^Other scheduled oral medications included atorvastatin 20 mg/day, benztropine 0.5 mg/day, clozapine 700 mg/day, docusate 250 mg/day, and propranolol 80 mg/day.

^24^Other scheduled oral medications included atorvastatin 20 mg/day, benztropine 1 mg/day, clozapine 600 mg/day, docusate 250 mg/day, and propranolol 80 mg/day.

^25^Other scheduled oral medications included atorvastatin 20 mg/day, benztropine 1 mg/day, clozapine 700 mg/day, docusate 250 mg/day, and propranolol 80 mg/day.

**Table 3:** VPA C/D ratio in Case 2.

Day^1^	Formulation	VPA dose^1^ (mg/day)	Concentration (*μ*g/mL)	C/D ratio	C/D ratio × 1000^2^
All 10 VPA concentrations (C/D ratio × 1000: mean ± SD = 25 ± 5.6, range = 17–33)					
8 therapeutic^2^ VPA concentrations (C/D ratio × 1000: mean ± SD = 24 ± 5.6, range = 17–33)					
5^3^	ECDVNa	1500	39	0.026	26
8^3^	ECDVNa	1500	49	0.033	33
14^4^	ECDVNa	2500	61	0.024	24
20^5^	ECDVNa	2500	75	0.030	30
23^6^	ECDVNa	2500	82	0.033	33
29^7^	ECDVNa	3000	77	0.026	26
36^7^	Concentrate	3500	72	0.020	20
41^7^	Concentrate	3500	78	0.022	22
48^8^	Concentrate	4000	73	0.018	18
55^9^	ECDVNa	4000	67	0.017	17

C/D: concentration-to-dose; ECDVNa: enterocoated divalproex sodium; VPA: valproic acid.

^1^The Pearson correlation between day of admission and VPA dose was *r* = 0.97 (*p* < 0.001); VPA dose remained significant in a partial correlation while controlling for VPA concentration: *r* = 0.96 (*p* < 0.001). This is compatible with a progressive autoinduction indicating, at least with dose range, that it was necessary to increase the dose as the duration lengthened.

^2^50–125 *μ*g/mL concentrations are considered therapeutic concentrations. As this patient has 2 nontherapeutic concentrations, it would be better to compare a priori with other patients using only the VPA therapeutic concentrations. In reality, eliminating the first 2 VPA concentrations, which were subtherapeutic, made almost no difference.

^3^Other scheduled oral medications included asenapine 10 mg/day, naproxen 1000 mg/day, omeprazole 20 mg/day, trimethoprim/sulfamethoxazole 1600/320 mg/day (for skin breakdown over swollen lower extremities), and trazodone 100 mg/day.

^4^Other scheduled oral medications included asenapine 15 mg/day, gabapentin 900 mg/day, lisinopril 10 mg/day, meloxicam 15 mg/day, metoprolol 50 mg/day, omeprazole 20 mg/day, trimethoprim/sulfamethoxazole 1600/320 mg/day (for skin breakdown over swollen lower extremities), tramadol 150 mg/day, and trazodone 150 mg/day.

^5^Other scheduled oral medications included asenapine 15 mg/day, gabapentin 900 mg/day, lisinopril 10 mg/day, meloxicam 15 mg/day, metoprolol 50 mg/day, omeprazole 20 mg/day, tramadol 150 mg/day, and trazodone 150 mg/day.

^6^Other scheduled oral medications included asenapine 15 mg/day, furosemide 40 mg/day, gabapentin 900 mg/day, lisinopril 10 mg/day, metoprolol 50 mg/day, omeprazole 20 mg/day, tramadol 150 mg/day, and trazodone 150 mg/day.

^7^Other scheduled oral medications included asenapine 15 mg/day, furosemide 20 mg/day, gabapentin 1200 mg/day, lisinopril 10 mg/day, metoprolol 50 mg/day, omeprazole 20 mg/day, tramadol 200 mg/day, and trazodone 150 mg/day.

^8^Other scheduled oral medications included asenapine 15 mg/day, furosemide 20 mg/day, gabapentin 1200 mg/day, metoprolol 50 mg/day, omeprazole 20 mg/day, tramadol 200 mg/day, and trazodone 150 mg/day.

^9^Other scheduled oral medications included asenapine 15 mg/day, furosemide 20 mg/day, gabapentin 1200 mg/day, metoprolol 50 mg/day, omeprazole 20 mg/day, polyethylene glycol 17 g/day, tramadol 200 mg/day, and trazodone 150 mg/day.

**Table 4:** VPA C/D ratio in Case 3.

Day^1^	VPA					Phenytoin	
	Formulation	Dose^1^ (mg/day)	Concentration (*μ*g/mL)	C/D ratio	C/D ratio × 1000	Dose (mg/day)	Concentration (*μ*g/mL)
All 137 VPA concentrations (C/D ratio × 1000: mean ± SD = 8 ± 3.5, range = 3–20)							
70 therapeutic^2^ VPA concentrations (C/D ratio × 1000: mean ± SD = 9 ± 3.4, range = 5–18)							
1^3^	ECDVNa	3375	46	0.014	14	500	15
33^3^	ECDVNa	3375	59	0.017	17	500	18
62^3^	ECDVNa	3375	43	0.013	13	500	16
86^3^	Concentrate	3350	22	0.007	7	500	
117^3^	ECDVNa	3450	43	0.012	12	500	11
147^3^	ECDVNa	3375	38	0.011	11	500	10
162^3^	Concentrate	3450	9	0.003	3	500	
174^3^	Concentrate	3450	28	0.008	8	500	18
177^3^	Concentrate	3450	31	0.009	9	500	18
189^3^	ECDVNa	3375	36	0.011	11	500	
202^3^	ECDVNa	3750	40	0.011	11	500	10
208^3^	ECDVNa	4125	40	0.010	10	500	14
212^3^	ECDVNa	4500	68	0.015	15	500	15
244^3^	ECDVNa	4500	61	0.014	14	500	15
274^3^	ECDVNa	4500	67	0.015	15	500	15
307^3^	ECDVNa	4500	46	0.010	10	500	23
310^3^	ECDVNa	4500	50	0.011	11	0	23
313^3^	ECDVNa	4500	39	0.009	9	500	10
316^3^	ECDVNa	4500	81	0.018	18	500	11
320^3^	ECDVNa	4500	31	0.007	7	500	15
324^3^	ECDVNa	4500	46	0.010	10	500	16
335^3^	ECDVNa	5000	64	0.013	13	500	19
350^3^	ECDVNa	5000	75	0.015	15	500	24
398^3^	ECDVNa	5000	52	0.010	10	460	16
400^3^	ECDVNa	5000	85	0.017	17	460	11
430^3^	ECDVNa	5000	59	0.012	12	460	16
439^3^	ECDVNa	5000	65	0.013	13	460	18
470^3^	ECDVNa	5000	54	0.011	11	460	16
502^3^	ECDVNa	5000	53	0.011	11	460	20
530^4^	ECDVNa	5000	77	0.015	15	460	17
559^4^	ECDVNa	5000	50	0.010	10	460	13
590^5^	ECDVNa	5000	48	0.010	10	460	12
621^6^	ECDVNa	5000	37	0.007	7	460	10
635^6^	ECDVNa	5000	39	0.008	8	460	11
646^6^	ECDVNa	5250	35	0.007	7	460	13
681^7^	ECDVNa	5250	37	0.007	7	460	8
713^7^	ECDVNa	5250	46	0.009	9	460	7
734^7^	ECDVNa	5250	40	0.008	8	460	10
762^7^	ECDVNa	6000	53	0.009	9	460	9
770^7^	ECDVNa	6000	43	0.007	7	460	8
798^7^	ECDVNa	6000	23	0.004	4	460	9
806^7^	ECDVNa	6000	32	0.005	5	460	10
817^7^	ECDVNa	7500	36	0.005	5	200	9
831^7^	ECDVNa	7500	43	0.006	6	460	5
838^7^	ECDVNa	7500	47	0.006	6	460	15
846^7^	ECDVNa	7500	24	0.003	3	460	10
852^7^	ECDVNa	7500	35	0.005	5	460	8
860^7^	ECDVNa	7500	51	0.007	7	460	
866^7^	ECDVNa	7500	42	0.006	6	460	3
888^7^	ECDVNa	7500	71	0.009	9	460	20
890^7^	ECDVNa	7500	81	0.011	11	460	20
916^7^	ECDVNa	7500	45	0.006	6	460	7
919^7^	ECDVNa	7500	46	0.006	6	460	7
929^7^	ECDVNa	8250	43	0.005	5	460	
943^7^	ECDVNa	9000	43	0.005	5	460	
950^7^	ECDVNa	9000	50	0.006	6	460	
957^7^	ECDVNa	9000	75	0.008	8	460	
964^7^	ECDVNa	9000	37	0.004	4	460	
971^7^	ECDVNa	9000	28	0.003	3	460	
972^7^	ECDVNa	9000	44	0.005	5	460	8
979^7^	ECDVNa	9000	64	0.007	7	460	6
985^7^	ECDVNa	9000	38	0.004	4	460	9
989^7^	ECDVNa	9000	50	0.006	6	460	8
992^7^	ECDVNa	9000	59	0.007	7	460	9
999^7^	ECDVNa	9000	54	0.006	6	460	6
1014^7^	ECDVNa	9000	35	0.004	4	460	6
1020^7^	ECDVNa	9000	42	0.005	5	460	5
1029^7^	ECDVNa	10500	43	0.004	4	460	7
1035^7^	ECDVNa	10500	102	0.010	10	460	6
1039^7^	ECDVNa	10500	54	0.005	5	460	1
1042^7^	ECDVNa	10500	77	0.007	7	460	6
1056^7^	ECDVNa	10500	120	0.011	11	460	6
1062^7^	ECDVNa	10500	39	0.004	4	460	8
1064^7^	ECDVNa	10500	47	0.004	4	460	7
1070^7^	ECDVNa	10500	56	0.005	5	460	6
1080^7^	ECDVNa	10500	51	0.005	5	460	10
1091^7^	ECDVNa	10500	54	0.005	5	460	11
1098^7^	ECDVNa	10500	63	0.006	6	460	12
1105^7^	ECDVNa	10500	49	0.005	5	460	12
1112^7^	ECDVNa	10500	47	0.004	4	460	8
1119^7^	ECDVNa	10500	51	0.005	5	460	6
1133^7^	ECDVNa	10500	91	0.009	9	460	7
1148^7^	ECDVNa	10500	50	0.005	5	460	13
1163^7^	ECDVNa	10500	40	0.004	4	460	9
1168^7^	ECDVNa	10500	62	0.006	6	460	10
1175^7^	ECDVNa	10500	63	0.006	6	460	13
1182^7^	ECDVNa	10500	110	0.010	10	460	11
1184^7^	ECDVNa	10500	61	0.006	6	460	10
1189^7^	ECDVNa	10500	56	0.005	5	460	10
1196^7^	ECDVNa	14000	133	0.010	10	460	9
1210^7^	ECDVNa	10500	67	0.006	6	460	13
1217^7^	ECDVNa	10500	65	0.006	6	460	14
1224^7^	ECDVNa	10500	87	0.008	8	460	11
1231^7^	ECDVNa	10500	70	0.007	7	460	13
1245^7^	ECDVNa	10500	65	0.006	6	460	15
1252^7^	ECDVNa	10500	97	0.009	9	460	9
1259^7^	ECDVNa	10500	77	0.007	7	460	13
1266^7^	ECDVNa	10500	56	0.005	5	460	12
1273^7^	ECDVNa	10500	97	0.009	9	460	14
1280^7^	ECDVNa	10500	60	0.006	6	460	16
1287^8^	ECDVNa	10500	39	0.004	4	460	19
1294^8^	ECDVNa	10500	103	0.010	10	460	15
1301^8^	ECDVNa	10500	48	0.005	5	460	19
1307^8^	ECDVNa	10500	72	0.007	7	460	13
1316^8^	ECDVNa	10500	54	0.005	5	460	13
1322^8^	ECDVNa	10500	71	0.007	7	460	13
1329^8^	ECDVNa	10500	75	0.007	7	460	13
1336^8^	ECDVNa	10500	98	0.009	9	460	12
1344^8^	ECDVNa	10500	93	0.009	9	460	11
1350^8^	ECDVNa	10500	65	0.006	6	460	15
1357^8^	ECDVNa	10500	58	0.006	6	460	23
1364^8^	ECDVNa	10500	73	0.007	7	460	18
1371^8^	ECDVNa	10500	106	0.010	10	460	24
1373^8^	ECDVNa	10500	106	0.010	10	460	20
1378^8^	ECDVNa	10500	89	0.008	8	460	15
1386^8^	ECDVNa	10500	97	0.009	9	460	17
1392^8^	ECDVNa	10500	72	0.007	7	460	21
1393^8^	ECDVNa	10500	87	0.008	8	460	23
1394^8^	ECDVNa	10500	122	0.012	12	230	22
1395^8^	ECDVNa	3500	37	0.011	11	0	20
1399^8^	ECDVNa	10500	130	0.012	12	400	8
1406^8^	ECDVNa	10500	94	0.009	9	400	10
1413^8^	ECDVNa	10500	126	0.012	12	400	12
1420^8^	ECDVNa	10500	108	0.010	10	400	12
1427^8^	ECDVNa	6000	59	0.010	10	400	21
1435^8^	ECDVNa	9000	110	0.012	12	400	5
1440^8^	ECDVNa	9000	86	0.010	10	400	8
1444^8^	ECDVNa	9000	82	0.009	9	400	10
1448^8^	ECDVNa	9000	147	0.016	16	400	10
1454^7^	ECDVNa	7000	93	0.013	13	400	14
1457^9^	ECDVNa	7000	138	0.020	20	400	11
1461^10^	ECDVNa	7000	72	0.010	10	400	13
1470^11^	ECDVNa	7000	101	0.014	14	400	13
1478	ECDVNa	7000	113	0.016	16	400	7
1483	ECDVNa	7000	97	0.014	14	400	6

C/D: concentration-to-dose; ECDVNa: enterocoated divalproex sodium; VPA: valproic acid.

^1^After day 1420, VPA dose was guided more by physical comfort than VPA concentrations; therefore, these latter days were eliminated to calculate the Pearson correlation between day of admission and VPA dose, which was *r* = 0.92 (*p* < 0.001); VPA dose remained very high in a partial correlation while controlling for VPA concentration: *r* = 0.89 (*p* < 0.001). This is compatible with a progressive autoinduction indicating, at least with dose range, that it was necessary to increase the dose as the duration lengthened. When all values (including those after day 1420) were used to calculate correlations, the *r* values were, not surprisingly, slightly reduced both for the total correlation of *r* = 0.83 (*p* < 0.001) and the partial correlation of *r* = 0.78 (*p* < 0.001).

^2^50–100 *μ*g/mL concentrations are considered therapeutic concentrations. As this patient has too many nontherapeutic concentrations, it may be better to compare with other patients using only the VPA therapeutic concentrations.

^3^Other scheduled oral medications included benztropine 4 mg/day, mesoridazine 200 mg/day, lorazepam 6 mg/day, and risperidone 12 mg/day.

^4^Other scheduled oral medications included benztropine 4 mg/day, lorazepam 6 mg/day, and risperidone 12 mg/day.

^5^Other scheduled oral medications included benztropine 4 mg/day, lorazepam 6 mg/day, and risperidone 4 mg/day.

^6^Other scheduled oral medications included benztropine 4 mg/day and lorazepam 6 mg/day.

^7^Other scheduled oral medications included lorazepam 6 mg/day.

^8^Other scheduled oral medications included lorazepam 6 mg/day and trazodone 150 mg/day.

^9^Other scheduled oral medications included lorazepam 4 mg/day.

^10^Other scheduled oral medications included lorazepam 3 mg/day.

^11^Other scheduled oral medications included lorazepam 1 mg/day.
